# 非小细胞肺癌LVD、MVD、CEAmRNA及KAI1和Kiss-1的表达与患者预后关系的研究

**DOI:** 10.3779/j.issn.1009-3419.2012.06.05

**Published:** 2012-06-20

**Authors:** 国文 汪, 祖义 王, 传奎 黎, 萍 王, 大敏 柴, 泽农 承

**Affiliations:** 1 233004 安徽，蚌埠医学院第一附属医院胸心外科 Department of Cardiothoracic Surgery, the First Affiliated Hospital of Bengbu Medical College, Bengbu 233004, China; 2 233004 安徽，蚌埠医学院第一附属医院病理科 Department of pathology, the First Affiliated Hospital of Bengbu Medical College, Bengbu 233004, China

**Keywords:** 肺肿瘤, 癌胚抗原, 肿瘤转移抑制基因, KAI1, Kiss-1, 预后, Lung neoplasms, Carcinoembryonic antigen, Tumor metastasis suppressor gene, KAI1, Kiss-1, Prognosis

## Abstract

**背景与目的:**

目前对非小细胞肺癌（non-small cell lung cancer, NSCLC）患者预后的研究很多，有些临床病理因素已被公认与预后有关，本研究旨在对淋巴管密度（lymphatic vessel density, LVD）、微血管密度（microvessel density, MVD）及癌胚抗原信使核糖核酸（carcinoembryonic antigen messenger ribonucleic acid, CEAmRNA）和肿瘤转移抑制基因*KAI1*、*Kiss-1*的表达与NSCLC患者预后的关系进行探讨。

**方法:**

选取57例NSCLC患者，采用逆转录巢式聚合酶链反应（nested-RT-PCR）和微流控芯片（micro-fluid chip）技术对外周血CEAmRNA的表达进行检测；采用免疫组织化学技术对肺癌组织LVD、MVD和KAI1、Kiss-1的表达进行检测，并对所有患者进行随访分析。

**结果:**

全组的5年生存率为18%，中位生存期为34个月；单因素分析显示TNM分期、淋巴结有无转移、CEAmRNA的阳性表达、MVD、LVD和Kiss-1的表达对生存有影响；多因素分析显示TNM分期、淋巴结有无转移和CEAmRNA的表达是影响预后的独立危险因素。

**结论:**

在NSCLC患者中MVD、LVD和Kiss-1及CEAmRNA的表达可以在一定程度上反映患者的预后。

## Abstract (en)

**Background and objective:**

In recent years, many studies have revealed the prognosis in patients with non-small cell lung cancer (NSCLC). In general, some clinic-pathological parameters have been related with prognosis. The aim of this study is to detect the relationship among lymphatic vessel density (LVD), microvessel density (MVD), expression of carcinoembryonic antigen (CEA) mRNA, metastasis suppressor genes (*KAI1* and *Kiss-1*), and the prognosis of NSCLC patients.

**Methods:**

Blood samples were collected from 57 cases of NSCLC. The transcription of CEA mRNA was detected via nested reverse transcriptase-polymerase chain reaction and micro-fluid chip. Immunohistochemistry was performed to detect the expression of LVD, MVD, KAI1 and Kiss-1 in the patients. All follow-up data were collected and analyzed.

**Results:**

The overall five-year survival rate was 18%, and the median survival was 34 months. TNM stage, lymph node metastasis, and expression of MVD, LVD, CEA mRNA and Kiss-1 were factors to survival, as determined via single survival analysis. Multivariate analysis demonstrated that TNM stage, lymph node metastasis, and expression of CEA mRNA were independent prognostic factors for NSCLC patients.

**Conclusion:**

The expression of MVD, LVD, Kiss-1 and CEA mRNA is related to the prognosis of NSCLC.

肺癌已成为癌症死亡的主要原因，每年大约有120万人死于肺癌，其中非小细胞肺癌（non-small cell lung cancer, NSCLC）占肺癌的80%以上^[1]^。其预后主要由分期决定，同时不同的分期也决定了治疗方案的选择^[2]^。NSCLC总的5年生存率相对较差，对于外科切除术后的患者5年生存率从Ⅰa期的70%到Ⅲa期的25%，大部分都是死于肿瘤的转移复发。同一分期不同的生存情况提示有影响预后的其它因素存在^[2]^。本文通过对NSCLC预后的影响因素进行分析，包括各临床病理因素及微血管密度（microvessel density, MVD）、淋巴管密度（lymphatic vessel density, LVD）、外周血癌胚抗原（carcinoembryonic antigen messenger ribonucleic acid, CEAmRNA）和转移抑制基因KAI1和Kiss-1的表达情况，探讨影响肺癌预后的可能因素，以期对NSCLC的综合治疗提供有意义的指导。

## 1 材料与方法

### 1.1 研究对象和标本采集

收集蚌埠医学院第一附属医院胸外科2005年12月-2006年8月行手术治疗的NSCLC病例共60例，剖胸探查1例，操作中标本破坏2例，实际入选57例。57例中男性46例，女性11例；鳞癌36例，腺癌16例，支气管肺泡细胞癌5例；术后pTNM分期Ⅰ期-Ⅳ期分别为17例、12例、23例和5例；伴淋巴结转移29例。检测LVD和MVD及KAI1、Kiss-1的表达所选标本为石蜡组织切片；检测CEAmRNA的表达所选标本为术前明确诊断的NSCLC患者外周血。

### 1.2 检测方法

#### 1.2.1 MVD检测

所选试剂主要有鼠抗人CD31单克隆抗体（克隆号JC/70A）、鼠抗人CD34单克隆抗体（克隆号QBEnd/10）及高温修复液和Elivision^TM^ plus Polyer HRP（Mouse/Rabbit）IHC Kit（福州迈新生物技术开发有限公司）。具体方法见参考文献^[3]^。

#### 1.2.2 LVD检测

##### 1.2.2.1 试剂及染色方法

鼠抗人单克隆抗体Podoplanin（18H5）购于美国Santa Cruz公司，Elivision二步法试剂盒为丹麦DAKO公司产品，购于福州迈新生物技术开发有限公司。实验方法采用免疫组织化学Elivision二步法。标本均予10%福尔马林固定，石蜡包埋，4 µm连续切片，脱蜡，脱二甲苯，水化。实验程序按试剂盒说明书进行。

##### 1.2.2.2 结果判定

结果判定采用盲法，每张切片由两名病理科专家分别计数。Podoplanin判定标准视内皮细胞形成的条状、腔隙状等的孤立或成簇状结构，棕黄染色的管腔为淋巴管，管腔 < 8个红细胞大小按一条淋巴管计数。Podoplanin染色阳性LVD计数方法：先于低倍光镜（40倍和100倍）下确定5个淋巴管着色最密集的区域，然后在400倍视野下计数淋巴管，取5个视野的均值作为LVD，两名医师计数差10%以上者重新计数。

#### 1.2.3 Kiss-1和KAI1检测

##### 1.2.3.1 试剂及染色方法

兔抗人多克隆抗体Kiss-1（FL-145）和兔抗人KAI1（H-173）多克隆抗体购于美国Santa Cruz公司，Elivision二步法试剂盒购于福州迈新生物技术开发有限公司。实验方法采用免疫组织化学Elivision二步法。实验程序按试剂盒说明书进行。抗体工作浓度为1:100。每次用已知Kiss-1和KAI1阳性标本切片作为阳性对照，用PBS代替一抗作为阴性对照。

##### 1.2.3.2 结果判定

Kiss-1蛋白和KAI1蛋白为细胞质和/或细胞膜出现棕色颗粒者为阳性细胞，采用免疫反应积分标准即染色强度和阳性细胞百分比的乘积^[4]^。阳性判定以染色强度及阳性细胞数综合判定。阳性细胞百分比：无细胞染色为0，1%-25%为1，26%-50%为2，51%-75%为3， > 75%为4。染色强度：不显色或显色不清为0，浅黄色为1，棕黄色为2，棕褐色为3。两分值乘积 > 3为阳性。

#### 1.2.4 CEAmRNA检测

采用一步法提取RNA，逆转录得cDNA，RT-PCR扩增，*Micro-fluid chip*方法进行检测，具体引物及方法见参考文献^[5]^。

### 1.3 统计分析

对全部病例进行随访，随访截止时间为2011年8月31日。数据处理采用SPSS 13.0软件，采用卡方检验、*t*检验对各临床病理因素进行分析；采用*Kaplan-Meier*法计算生存率，*Log-rank*检验比较生存率差异，*Cox*比例风险模型进行多因素生存分析。*P* < 0.05为差异有统计学意义。

## 2 结果

### 2.1 LVD、MVD、CEAmRNA及KAI1和Kiss-1的表达与临床病理因素之间的关系

见图 1、图 2、表 1、表 2。本文对外周血CEAmRNA的检测结果与临床病理之间的关系与以前研究结果大致一致，与TNM分期和淋巴结转移有关^[5]^。按TNM分期把全组资料分为Ⅰ+Ⅱ组和Ⅲ+Ⅳ组，统计结果显示两组间LVD（*P*=0.001）、MVD（CD31:*P*=0.027; CD34:*P*=0.040）及肿瘤转移抑制基因KAI1（*P*=0.044）和Kiss-1（*P*=0.024）的表达差异具有统计学意义；按有无淋巴结转移把全组资料分为N0组和N1+N2组，统计结果显示两组间LVD（*P* < 0.001）、MVD（CD31:*P*=0.002; CD34:*P*=0.013）及肿瘤转移抑制基因KAI1（*P*=0.011）和Kiss-1（*P* < 0.001）的表达差异具有统计学意义；LVD、MVD、CEAmRNA及肿瘤转移抑制基因KAI1和Kiss-1的表达与年龄、性别、部位、吸烟、病理类型及分化程度无明显关系。

**1:** 57例NSCLC患者LVD和MVD与各临床病理特征之间的关系 The relationship between LVD、MVD and clincopathological parameters

Characteristic	*n*	CD31	*t*	*P*		CD34	*t*	*P*		LVD	*t*	*P*
Sex			0.862	0.392			0.084	0.933			0.554	0.584
Male	46	28.72±11.0				35.02±12.71				16.76±7.15		
Female	11	31.82±9.25				35.36±9.00				17.64±3.91		
Age (year)			0.224	0.824			0.208	0.836			0.232	0.817
> 55	37	29.08±11.68				35.30±13.87				17.08±6.58		
≤55	20	29.75±8.84				34.70±7.80				16.65±6.87		
Smoking			0.007	0.995			0.289	0.773			0.551	0.584
Yes	34	29.32±10.90				34.71±12.87				16.53±6.16		
No	23	29.30±10.61				35.65±10.88				17.52±7.37		
Location			0.132	0.895			0.605	0.547			0.950	0.346
Center	42	29.43±11.16				35.67±12.60				17.43±6.69		
Periphery	15	29.00±9.58				33.47±10.43				15.53±6.47		
Histologic classification			0.052	0.959			0.981	0.331			0.935	0.354
Squamous cancer	32	29.25±12.01				36.47±13.91				17.66±6.96		
Non-squamous cancer	25	29.40±8.96				33.32±9.00				16.00±6.19		
Differentiation			1.168	0.248			1.034	0.306			0.287	0.775
Moderate+well	39	30.44±10.26				36.21±11.24				17.10±6.22		
Poor	18	26.89±11.48				32.67±13.57				16.56±7.63		
Stage			2.271	0.027			2.106	0.040			3.566	0.001
Ⅰ+Ⅱ	34	26.76±10.56				32.41±11.66				14.59±6.45		
Ⅲ+Ⅳ	23	33.09±10.93				39.04±11.67				20.39±5.32		
Lymph node metastasis			3.239	0.002			2.553	0.013			6.025	< 0.001
N0	28	25.00±8.93				31.14±10.65				12.71±4.78		
N1+N2	29	33.48±10.73				38.90±12.20				21.00±5.56		

**2:** 57例NSCLC患者KAI1、Kiss-1和CEAmRNA与各临床病理特征之间的关系 The relationship between the rates of KAI1、Kiss-1、CEAmRNA and clincopathological parameters

Characteristic	*n*	Kiss-1					KAI1					CEAmRNA			
		Positive	Negative	*χ*^2^	*P*		Positive	Negative	*χ*^2^	*P*		Positive	Negative	*χ*^2^	*P*
Sex				0.315	0.575				0.160	0.689				1.919	0.166
Male	46	21	25				26	20				20	26		
Female	11	4	7				3	8				5	6		
Age (year)				0.187	0.665				0.426	0.514				1.094	0.296
＞55	37	17	20				20	17				15	22		
≤55	20	8	12				9	11				11	9		
Smoking				0.352	0.553				2.143	0.143				0.657	0.418
Yes	34	16	18				20	14				17	17		
No	23	9	14				9	14				9	14		
Location				0.124	0.725				0.682	0.409				0.009	0.924
Center	42	19	23				20	22				23	19		
Periphery	15	6	9				9	6				7	8		
Histologic classification				1.125	0.289				0.148	0.701				0.568	0.451
Squamous cancer	32	16	16				17	15				16	16		
Non-squamous cancer	25	9	16				12	13				10	10		
Differentiation				1.457	0.227				0.231	0.631				0.483	0.487
Moderate+well	39	15	24				19	20				19	20		
Poor	18	10	8				10	8				7	11		
Stage				5.092	0.024				4.047	0.044				17.394	< 0.001
Ⅰ+Ⅱ	34	19	15				21	13				8	26		
Ⅲ+Ⅳ	23	6	17				8	15				18	5		
Lymph node metastasis				13.423	< 0.001				6.474	0.011				9.726	0.002
N0	28	19	9				19	9				7	21		
N1+ N2	29	6	23				10	19				19	10		

**1:**
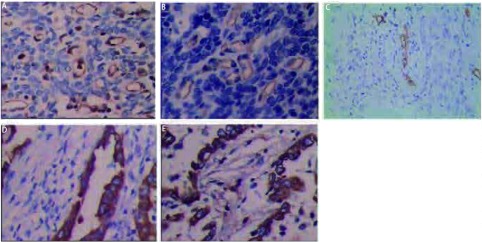
LVD、MVD计数和KAI1、Kiss-1的阳性表达（免疫组化，×400）。A：微血管密度（CD31）；B：微血管密度（CD34）；C：淋巴管密度；D：肺腺癌KAI1；E：肺腺癌Kiss-1。 The counts of LVD, MVD and positive expression of KAI1, Kiss-1 (Immunohistochemistry, ×400). A: The microvessel density (CD31); B: The microvessel density(CD34); C: The lymphatic vessel density; D: KAI1 in adenocarcinoma; E: Kiss-1 in adenocarcinoma.

**2:**
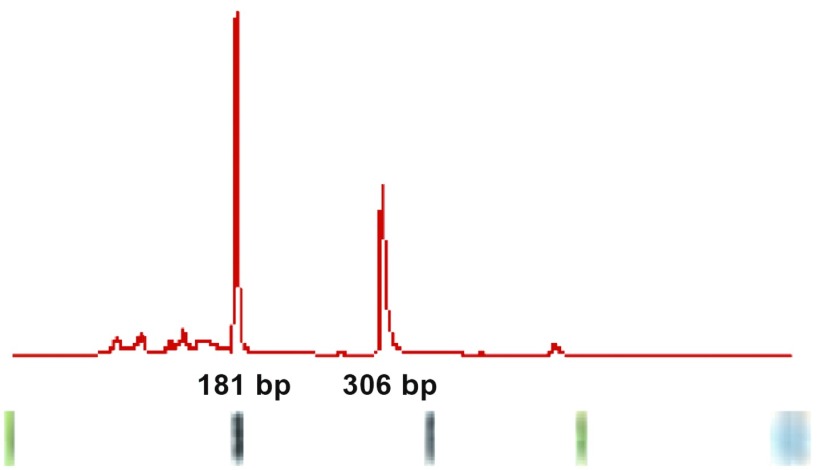
上方是CEA mRNA和GAPDH mRNA扩增产物的波峰曲线，下方是对应的电泳图 The upper panel showing ridge curves of amplification of CEA mRNA and GAPDH mRNA, and the lower panel showing the electropherograms of the same samples (basal line of ridge is 0.5)

### 2.2 生存影响因素分析

全组57例患者随访时间为3个月-81个月，随访率为100%。全组中位生存期为34个月，1年、3年、5年生存率分别为60%、32%和18%，；单因素分析显示TNM分期（*P* < 0.001）、淋巴结有无转移（*P* < 0.001）、CEAmRNA（*P* < 0.001）的阳性表达、MVD（*P*=0.001）、LVD（*P* < 0.001）和Kiss-1（*P*=0.001）的表达对生存有影响（图 3）。多因素分析显示TNM分期（*P* < 0.001）、淋巴结有无转移（*P* < 0.001）和CEAmRNA（*P* < 0.001）的表达是影响预后的独立危险因素（表 3）。

**3:** 57例NSCLC患者的多因素生存分析 Multivariate survival analysis of 57 NSCLC cases

Item	B	SE	Wald	*P*	OR	95%Cl for Exp(B)	
						Lower	Upper
Lymph node metastasis	1.947	0.483	16.264	< 0.001	7.077	2.720	18.048
TNM stage	1.699	0.485	12.270	< 0.001	5.469	2.114	14.152
CEA mRNA	2.900	0.548	27.989	< 0.001	18.179	6.280	53.237

**3:**
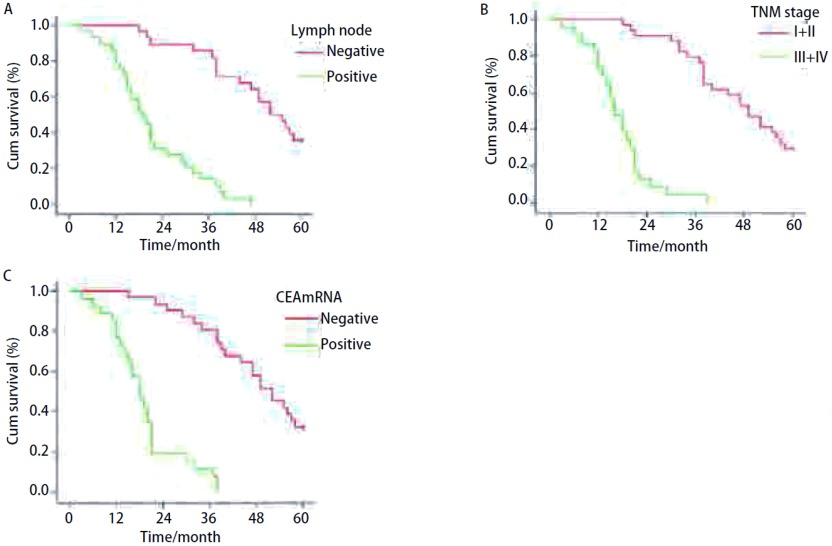
生存曲线。A：淋巴结转移（*P* < 0.001）；B：TNM分期（*P* < 0.001）；C：CEA mRNA表达（*P* < 0.001）。 Cumulatiive survival time curves. A: Negative and positive lymphatic node (*P* < 0.001); B: Different TNM stages (*P* < 0.001); C: CEA mRNA positive and negative expression (*P* < 0.001).

## 3 讨论

肺癌的治疗水平在提高，但近十年肺癌患者的生存率几乎没有改变，快速而广泛的转移使得对肺癌患者的治疗远不能令人满意^[6, 7]^。肺癌的TNM分期被广泛地用于评价预后，但即使行根治性切除的Ⅰ期NSCLC仍有大约30%的患者最终死于肿瘤的复发。对于术后的患者，虽然病理分期是影响预后最重要的因素，但由于肿瘤生物学行为的差异使同一分期的不同患者预后差异也很大^[8]^。因此，要进一步提高肺癌的生存率，就必须寻找新的预后影响因素，从而从不同的途径干预肺癌的进展，以获得更好的治疗效果。

癌的发生是一个多阶段的过程，包括自主增殖信号的获得、对生长抑制信号的不敏感、对凋亡信号的耐受、持续的血管发生、侵袭和转移6个必要的步骤^[9, 10]^。本文主要是对影响最后两个阶段的标记物进行分析，以期获得能较好反应预后的标记物，从而在一定程度上指导临床治疗。在肿瘤的侵袭和转移过程中血管的生成是一个不可或缺的环节。它不仅为肿瘤细胞提供血供，而且也是转移的重要途径，而血管内皮细胞染色可以清楚地显示肿瘤的血管密度，反应肿瘤的生物学特性。常用于血管内皮细胞染色的试剂有CD31、CD34、CD105、FⅧ等。Meert^[11]^和Mineo^[12]^等研究发现MVD与NSCLC的预后呈负相关。本项研究显示MVD与预后有关（*P*=0.001），但*Cox*生存分析显示其不是影响预后的独立危险因素。因此，我们认为肿瘤的血管生成有利于肿瘤的生长和经血行进行转移，但其对预后的影响与肿瘤细胞自身的侵袭性有关，包括肿瘤细胞穿透血管内皮进入血液循环的能力。

淋巴转移是肿瘤转移的重要方式之一，也是判断患者预后的重要因素。Kadota等^[13]^研究了微小淋巴管、管状淋巴管和总淋巴管密度与患者预后之间的关系，结果显示微小淋巴管密度是影响预后的因素。王艳等^[14]^研究显示高淋巴管密度是NSCLC患者有意义的、独立的不良预后因素。本项研究表明，以Podoplanin为标记物的LVD是影响预后有意义的风险因素，同相关研究结果一致。作为组织液回收的途径，淋巴管的生成能增加肿瘤细胞经淋巴管进行转移的几率，作为主动引流的淋巴管，其密度可能直接关系到患者的转移并进而影响预后。

细胞遗传学改变可导致其生长调控失衡使细胞增殖失去控制，但无限制的生长并不能引起癌细胞的浸润和转移，后者的表型还需要转移抑制基因功能缺失等分子事件^[15]^。在对胰腺癌、口腔癌、宫颈癌、结肠癌及头颈癌等多种实体瘤的研究中都发现肿瘤抑制基因KAI1基因表达下调，在伴有淋巴结转移的癌组织中表达较不伴有淋巴结转移的癌组织降低，在转移癌中的表达较原发癌降低更明显^[16]^。陈清勇等^[17]^研究显示在不同的肿瘤分期、分化程度和淋巴结有无转移中，KAI1/CD82的表达水平具有统计学差异。Kiss-1在部分肿瘤中表达缺失并与侵袭转移密切相关^[18]^。本研究显示在肿瘤的TNM分期和有无淋巴结转移中，KAI1及Kiss-1的表达具有统计学差异。同为转移抑制基因，分析显示Kiss-1的表达与预后相关，而KAI1的表达不能反映预后，是标本量的影响还是有其它内在机制的影响还需进一步研究。近期有研究表明^[19, 20]^，对肿瘤转移有促进作用的基因表达产物肿瘤转移相关蛋白1（MTA1）的表达是影响I期NSCLC患者预后的独立危险因素。

CEA是基因*CEACAM5*的产物，而该基因只位于各种黏膜上皮和上皮来源的癌细胞，因此在外周血检测到CEAmRNA则可表明其外周血循环中存在有癌细胞，并间接证明上皮来源的肿瘤已经出现血行微转移。已有研究^[21]^显示CEAmRNA是反映NSCLC微转移的一个可靠指标。Benlloch等^[22]^采用实时定量逆转录多聚酶链反应对纵隔淋巴结中CEACAM5的表达进行检测，结果显示其可以对淋巴结中微转移肿瘤细胞进行评价，与传统分期相比，对复发危险度的评估具有更高的精确度。本文对外周血CEAmRNA的检测结果与临床病理之间的关系与以前研究结果大致一致^[5]^，生存分析显示CEAmRNA的表达是影响预后有意义的风险因素。同时，本实验所用的微流控芯片技术与传统的琼脂糖凝胶电泳相比具有明显的优势^[23]^。

除了传统的TNM分期和淋巴结的转移情况，本实验结果显示MVD、LVD及Kiss-1和CEAmRNA的表达是影响生存的有意义因素，而*Cox*比例风险模型分析显示淋巴结的转移、TNM分期和CEAmRNA的表达是影响患者预后的独立危险因素，因此，在有条件的前提下术前对NSCLC患者进行该项检测并采取相应的治疗方案，有可能在一定程度上改善患者的预后。
